# Supplementary material for: Neural correlates of perceiving and interpreting engraved prehistoric patterns as human production: Effect of archaeological expertise
Source: PLoS One. 2022 Aug 3;17(8):e0271732. doi: 10.1371/journal.pone.0271732 (PMC9348741; doi:10.1371/journal.pone.0271732)
Supplement: S1 Table — (DOCX) [file pone.0271732.s001.docx]

**S1 Table**: Contextual and descriptive data on early engravings used as visual stimuli

| Site | Age (kyr BP) | Reference |
| --- | --- | --- |
| Blombos (South Africa) | 100–75 | (Henshilwood et al., 2009)  (d’Errico et al., 2001, p. 70000)  (d’Errico & Henshilwood, 2007) |
| Klasies River (South Africa) | 100-85 | (d’Errico et al., 2012) |
| Pinnacle Point (South Africa) | 100 | (Watts, 2010) |
| Kozarnika (Bulgaria) | 900 | (Guadelli & Guadelli, 2003) |
| Pešturina (Serbia) | 95-64 | (Majkić, d’Errico, Milošević, et al., 2018) |
| Quneitra (Israel) | 50 | (Marshack, 1996) |
| Kiik-Koba (Crimea) | 35-37 | (Majkić, d’Errico, & Stepanchuk, 2018) |
| Klipdrift Shelter (South Africa) | 65-59 | (Henshilwood et al., 2014) |

d’Errico, F., García Moreno, R., & Rifkin, R. F. (2012). Technological, elemental and colorimetric analysis of an engraved ochre fragment from the Middle Stone Age levels of Klasies River Cave 1, South Africa. *Journal of Archaeological Science*, *39*(4), 942–952. https://doi.org/10.1016/j.jas.2011.10.032

d’Errico, F., & Henshilwood, C. S. (2007). Additional evidence for bone technology in the southern African Middle Stone Age. *Journal of Human Evolution*, *52*(2), 142–163. https://doi.org/10.1016/j.jhevol.2006.08.003

D’Errico, F., Henshilwood, C., & Nilssen, P. (2001). An engraved bone fragment from c. 70,000-year-old Middle Stone Age levels at Blombos Cave, South Africa: Implications for the origin of symbolism and language. *Antiquity*, *75*(288), 309–318. https://doi.org/10.1017/S0003598X00060968

Guadelli, A., & Guadelli, J.-L. (2003). Une expression « symbolique » sur os dans le Paléolithique inférieur: Étude préliminaire de l’os incisé de la grotte Kozarnika, Bulgarie du Nord-Ouest. *« La spiritualité ». U.I.S.P.P. VIIIème Commission : Paléolithique supérieur*, *106*, 87–95, 7 fig., 3 tab. https://halshs.archives-ouvertes.fr/halshs-00197589

Henshilwood, C. S., d’Errico, F., & Watts, I. (2009). Engraved ochres from the Middle Stone Age levels at Blombos Cave, South Africa. *Journal of Human Evolution*, *57*(1), 27–47. https://doi.org/10.1016/j.jhevol.2009.01.005

Henshilwood, C. S., van Niekerk, K. L., Wurz, S., Delagnes, A., Armitage, S. J., Rifkin, R. F., Douze, K., Keene, P., Haaland, M. M., Reynard, J., Discamps, E., & Mienies, S. S. (2014). Klipdrift Shelter, southern Cape, South Africa: Preliminary report on the Howiesons Poort layers. *Journal of Archaeological Science*, *45*, 284–303. https://doi.org/10.1016/j.jas.2014.01.033

Majkić, A., d’Errico, F., Milošević, S., Mihailović, D., & Dimitrijević, V. (2018). Sequential Incisions on a Cave Bear Bone from the Middle Paleolithic of Pešturina Cave, Serbia. *Journal of Archaeological Method and Theory*, *25*(1), 69–116. https://doi.org/10.1007/s10816-017-9331-5

Majkić, A., d’Errico, F., & Stepanchuk, V. (2018). Assessing the significance of Palaeolithic engraved cortexes. A case study from the Mousterian site of Kiik-Koba, Crimea. *PLOS ONE*, *13*(5), e0195049. https://doi.org/10.1371/journal.pone.0195049

Marshack, A. (1996). A Middle Paleolithic Symbolic Composition From the Golan Heights: The Earliest Known Depictive Image. *Current Anthropology*, *37*(2), 357–365. https://doi.org/10.1086/204499

Watts, I. (2010). The pigments from Pinnacle Point Cave 13B, Western Cape, South Africa. *Journal of Human Evolution*, *59*(3–4), 392–411. https://doi.org/10.1016/j.jhevol.2010.07.006
