# Supplementary material for: Neural correlates of perceiving and interpreting engraved prehistoric patterns as human production: Effect of archaeological expertise
Source: PLoS One. 2022 Aug 3;17(8):e0271732. doi: 10.1371/journal.pone.0271732 (PMC9348741; doi:10.1371/journal.pone.0271732)
Supplement: S2 Table — (DOCX) [file pone.0271732.s002.docx]

Table S1 : Mean value and standard deviation of the BOLD signal in the 64 hROIs activated by at least one of the two groups of participants. in Attribution minus Orientation contrast.

| **BOLD signal in [Attribution *minus* Orientation]** | | | | | | | | |  |
| --- | --- | --- | --- | --- | --- | --- | --- | --- | --- |
|  | Controls | | | | Experts | | | | |
|  | Left hemisphere | | Right hemisphere | | Left hemisphere | | Right hemisphere | | |
|  | Mean  BOLD | SD | Mean BOLD | SD | Mean BOLD | SD | Mean BOLD | SD | |
| G_Cingulum_Ant-2 | - | - | 0.30 | 0.23 | - | - | 0.29 | 0.24 | |
| G_Cingulum_Mid-2 | 0.15 | 0.23 | 0.21 | 0.25 | 0.23 | 0.16 | 0.24 | 0.17 | |
| G_Cingulum_Post-1 | - | - | 0.15 | 0.21 | - | - | 0.05 | 0.30 | |
| G_Cingulum_Post-3 | 0.08 | 0.29 | - | - | 0.39 | 0.36 | - | - | |
| G_Frontal_Sup_Medial-3 | - | - | 0.20 | 0.20 | - | - | 0.26 | 0.25 | |
| G_Fusiform-1 | 0.16 | 0.12 | - | - | 0.09 | 0.12 | - | - | |
| G_Fusiform-2 | 0.20 | 0.17 | 0.14 | 0.16 | 0.11 | 0.13 | 0.06 | 0.09 | |
| G_Fusiform-3 | 0.13 | 0.17 | - | - | 0.15 | 0.15 | - | - | |
| G_Fusiform-4 | 0.29 | 0.36 | 0.16 | 0.22 | 0.28 | 0.21 | 0.19 | 0.15 | |
| G_Fusiform-5 | 0.33 | 0.18 | 0.30 | 0.21 | 0.28 | 0.19 | 0.21 | 0.17 | |
| G_Fusiform-6 | 0.30 | 0.24 | 0.32 | 0.24 | 0.25 | 0.19 | 0.21 | 0.17 | |
| G_Fusiform-7 | 0.27 | 0.35 | - | - | 0.19 | 0.21 | - | - | |
| G_Hippocampus-2 | 0.07 | 0.15 | 0.09 | 0.12 | 0.12 | 0.1 | 0.11 | 0.12 | |
| G_Insula-anterior-2 | 0.36 | 0.32 | 0.31 | 0.23 | 0.34 | 0.2 | 0.33 | 0.22 | |
| G_Insula-anterior-3 | 0.36 | 0.31 | 0.29 | 0.25 | 0.34 | 0.34 | 0.33 | 0.3 | |
| G_Occipital_Lat-2 | 0.40 | 0.46 | 0.38 | 0.41 | 0.4 | 0.3 | 0.41 | 0.27 | |
| G_Occipital_Lat-3 | 0.37 | 0.51 | 0.28 | 0.49 | 0.48 | 0.38 | 0.36 | 0.39 | |
| G_Occipital_Lat-4 | 0.51 | 0.39 | 0.55 | 0.4 | 0.36 | 0.28 | 0.43 | 0.32 | |
| G_Occipital_Lat-5 | 0.34 | 0.26 | 0.38 | 0.29 | 0.28 | 0.25 | 0.25 | 0.21 | |
| G_Occipital_Mid-1 | 0.16 | 0.27 | 0.16 | 0.17 | 0.17 | 0.26 | 0.17 | 0.17 | |
| G_Occipital_Mid-2 | 0.15 | 0.19 | 0.13 | 0.18 | 0.13 | 0.19 | 0.07 | 0.15 | |
| G_Occipital_Pole-1 | 0.36 | 0.47 | 0.39 | 0.66 | 0.54 | 0.33 | 0.43 | 0.36 | |
| G_ParaHippocampal-2 | - | - | 0.24 | 0.23 | - | - | 0.14 | 0.14 | |
| G_Supp_Motor_Area-1 | 0.27 | 0.40 | 0.32 | 0.31 | 0.21 | 0.19 | 0.34 | 0.34 | |
| N_Caudate-4 | 0.04 | 0.15 | - | - | 0.18 | 0.18 | - | - | |
| N_Caudate-5 | 0.21 | 0.30 | 0.19 | 0.38 | 0.33 | 0.28 | 0.45 | 0.34 | |
| N_Caudate-6 | - | - | 0.05 | 0.23 | - | - | 0.18 | 0.19 | |
| N_Thalamus-1 | 0.26 | 0.36 | 0.21 | 0.41 | 0.58 | 0.36 | 0.57 | 0.43 | |
| N_Thalamus-2 | -0.00 | 0.28 | 0.05 | 0.26 | 0.21 | 0.24 | 0.23 | 0.24 | |
| N_Thalamus-3 | 0.29 | 0.44 | - | - | 0.38 | 0.32 | - | - | |
| N_Thalamus-4 | 0.15 | 0.38 | 0.19 | 0.36 | 0.50 | 0.31 | 0.52 | 0.39 | |
| N_Thalamus-5 | 0.08 | 0.19 | 0.12 | 0.2 | 0.09 | 0.13 | 0.09 | 0.14 | |
| N_Thalamus-7 | - | - | 0.11 | 0.29 | - | - | 0.14 | 0.19 | |
| S_Cingulate-1 | 0.34 | 0.32 | 0.39 | 0.34 | 0.28 | 0.24 | 0.34 | 0.27 | |
| S_Cingulate-2 | 0.21 | 0.29 | 0.26 | 0.23 | 0.15 | 0.2 | 0.16 | 0.2 | |
| S_Inf_Frontal-1 | - | - | 0.36 | 0.31 | - | - | 0.23 | 0.30 | |
| S_Inf_Frontal-2 | - | - | 0.20 | 0.28 | - | - | 0.22 | 0.27 | |
| S_Intraoccipital-1 | - | - | 0.31 | 0.32 | - | - | 0.27 | 0.32 | |
| S_Orbital-1 | - | - | 0.15 | 0.21 | - | - | 0.10 | 0.15 | |
| S_Orbital-2 | - | - | 0.46 | 0.33 | - | - | 0.47 | 0.2 | |
| S_Precentral-4 | - | - | 0.06 | 0.18 | - | - | 0.09 | 0.14 | |
